# Fluid dynamics of life: exploring the physiology and importance of water in the critical illness

**DOI:** 10.3389/fmed.2024.1368502

**Published:** 2024-04-30

**Authors:** Henry Robayo-Amortegui, Alejandro Quintero-Altare, Catalina Florez-Navas, Isacio Serna-Palacios, Andrea Súarez-Saavedra, Ricardo Buitrago-Bernal, Julian Orlando Casallas-Barrera

**Affiliations:** ^1^Department of Critical Care Medicine, Fundación Clínica Shaio, Bogotá, DC, Colombia; ^2^Department of Medicine, Critical Care Resident, Universidad de La Sabana, Chía Cundinamarca, Colombia; ^3^Exploratorium group, Fundación Clínica Shaio, Bogotá, DC, Colombia

**Keywords:** water, critical care, homeostasis, biochemistry, human body

## Abstract

Water acknowledged as a vital component for life and the universal solvent, is crucial for diverse physiological processes in the human body. While essential for survival, the human body lacks the capacity to produce water, emphasizing the need for regular ingestion to maintain a homeostatic environment. The human body, predominantly composed of water, exhibits remarkable biochemical properties, playing a pivotal role in processes such as protein transport, thermoregulation, the cell cycle, and acid–base balance. This review delves into comprehending the molecular characteristics of water and its interactions within the human body. The article offers valuable insights into the intricate relationship between water and critical illness. Through a comprehensive exploration, it seeks to enhance our understanding of water’s pivotal role in sustaining overall human health.

## Background

"Water is peculiar. It's a liquid when it should be a gas, expands when it should contract, and dissolves almost everything it touches given enough time. Yet, without the peculiarity of water, the Earth would be just another lifeless snowball in space."

Yuan Lee

Water is considered one of the vital components for life, recognized as the universal solvent and the primary constituent of the human body. It is essential for most physiological processes necessary for survival. However, our body lacks the capacity to produce it, so it is necessary to acquire it through ingestion to maintain a homeostatic environment conducive to carrying out essential life processes, such as protein transport, thermoregulation, the cell cycle, and acid–base balance (1–3). Over time, our body has adapted to the terrestrial environment to ensure the intake of the necessary amount of water to carry out our, daily activities (2).

The human body is largely composed of water, playing a crucial role in numerous physiological processes due to its exceptional biochemical properties. In the clinical context, water plays a fundamental role in the formulation of numerous solutions used in medicine (1). Despite its critical importance in the care of critically ill patients, recent years have seen evidence pointing to potential adverse effects associated with excessive administration of fluids or non-physiological solutions, especially concerning the electrolytes present in these solutions (4, 5). This phenomenon has sparked growing interest in exploring the molecular characteristics of water and its interaction with other molecules in the human body. This article provides a comprehensive review that delves into evolutionary processes, biochemical properties, and some physiological functions in the human body. Additionally, it explores their relationship to critical illness.

## Methodology

A narrative review was performed, information related to the topic was compiled from the following databases: PubMed, Embase, Ovid, Ovid Books, Clinical Key, and Ebooks. A search was conducted using the MESH terms: Water AND homeostasis AND biochemistry AND Human body AND critical care, and the results in both English and Spanish languages were included. Articles that matched the criteria from the year 1990 to 2024 were incorporated, encompassing various types of articles, including systematic reviews, books, documents, narrative reviews, and research articles.

## Discussion

### Theories on the origin of water and the emergence of the first life forms

"The water is the element and principle of things."

"All things are made of water, and all things dissolve into water."

Thales of Miletus

The term ‘water’ encompasses any species containing hydrogen (H) and forming various states of water molecules through chemical reactions, including hydroxyl groups and protons bound in minerals (6). Although it constitutes most of our planet, covering two-thirds of its surface, the origin of this molecule remains uncertain (7). Various theories suggest that water might have arisen from condensed ice in planetesimals, small celestial bodies that gravitationally merged to form planets (8, 9). These sources of solid-state water, exposed to intense ultraviolet (UV) radiation, could have contributed to thawing and the formation of hydrothermal vents in active marine volcanoes, potentially indicating the origin of water and the formation of oceans (10). Seawater primarily contains chlorine (Cl-) 540 meq/L, sodium (Na+) 460 meq/L, magnesium (Mg+) 50 meq/L, sulfur oxide (SO) 30 meq/L, calcium (Ca+) 10 meq/L, and potassium (K+) 10 meq/L, suggesting that early chemical reactions took place in saline-rich environments (2, 11).

The first cells formed a lipid barrier, thus establishing an aqueous environment with a balanced acid–base equilibrium, ionic forces, and pressure, providing the ideal conditions for protein synthesis and biochemical reactions (8). Lipid membranes have played a critical role in the development of more sophisticated cellular structures. This has led to the creation of organs, enabling communication between them for the proper functioning of the human body (7, 8).

The evolution of early living systems is inherently linked to the role of water, as originally proposed by Claude Bernard: Cells are immersed in a fluid referred to as the ‘internal environment,’ which is now known as extracellular fluid (2, 9). In fact, the composition of the human body’s internal environment shares similarities with the electrolytes presents in the sea (2, 11). This internal environment is further subdivided into intracellular and extracellular spaces, defining it as the aqueous compartment of the organism where vital biochemical processes take place (2, 11).

Throughout the process of evolution, living organisms have developed adaptations to survive in environments with limited water resources. For instance, *Australopithecus* and *Homo erectus* increased their body surface area to enhance heat dissipation and reduce exposure to solar radiation, allowing them to reduce their water requirements by up to 18% (2, 12). Consequently, human anatomy has undergone phenotypic changes, such as variations in skin color, hair type, and nose shape, as well as adjustments in physical activity to regulate heat loss. These adaptations are aimed at fulfilling the various physiological processes necessary for the survival of the species and adapting to the environment. In this context, as proposed by Delpire et al., we could consider ourselves as “walking bags of water on the Earth’s surface” (2, 13–15).

### Biochemical structure and its significance

"…The importance of hydrogen bonding in physiology is greater than that of any other structural feature."

Linus Pauling

While water is the most common liquid on planet Earth, its understanding and the complete process of how this molecule is created are far from complete. In fact, water is considered the ultimate universal solvent, being the only substance capable of existing in liquid, solid, and gaseous forms. Its significance lies in its contribution to a variety of biomolecular processes essential for the human body (1). When compared to other fluids, water reveals its true uniqueness. To fully understand its function in the body and its interaction with other molecules, it is essential to explore its molecular structure. Each water molecule is composed of the covalent bonding of one oxygen (O) atom with two H-atoms. O-atom has 8 electrons, with its outer orbital consisting of 6 electrons, while H-atom has 1 electron. To satisfy the octet rule, O-atom attracts 2 electrons provided by 2 H-atoms. This results in a negative electrical charge on the O (O2-) and a positive electrical charge on the H (H+) atoms, which have donated their electrons (1, 16, 17).

**Figure 1:**
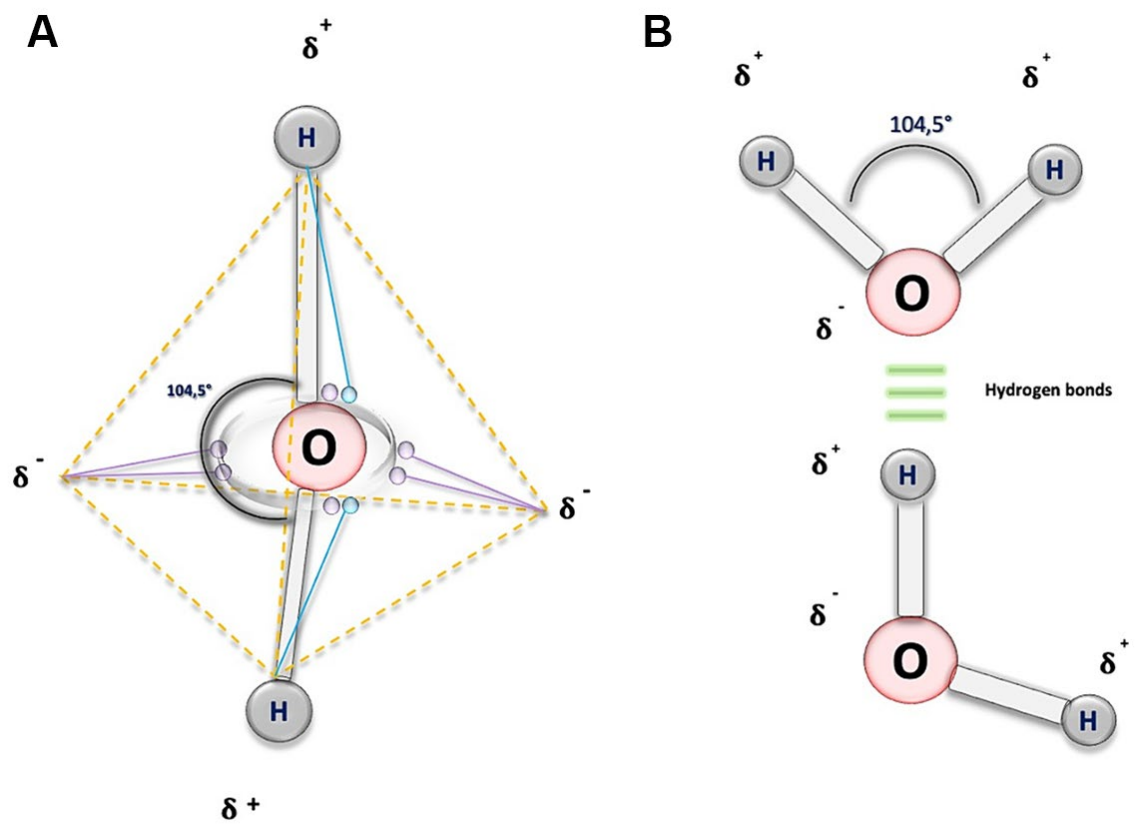
Biochemical structure of water molecule. **(A)** This illustration depicts a water molecule by representing an atom of oxygen (O), and purple circles, symbolizing the six electrons in the outermost orbital shell. To adhere to the octet rule, each hydrogen (H) atom shares its electron (blue circles) with O, resulting in a change in the electrical charge of the atoms. O acquires two partial negative charges, indicated by the symbol 𝛅-, while the H exhibit partial positive charges, denoted by 𝛅+. The dashed orange lines outline the tetrahedral geometry of the molecule, and the continuous grey lines represent the covalent bonds forming an angle of 104.5 degrees. **(B)** In this figure, the O atoms are linked by covalent bonds (depicted by gray bars) to the H atoms, shown as gray circles. The 104.5-degree angle is maintained, depicted by a black curved line. Additionally, the three horizontal green lines symbolize H bonds, interactions that occur between the negative electrical charges of O and the positive electrical charges of the H.

From a geometric perspective, the structure of water is determined by the outer electronic orbitals that originate from the bonds with H-atoms. As a result, when two H-atoms bond with an O-atom, they form a bond angle of approximately 104.5 degrees (°), slightly less than the 109 ° it would have in a perfect tetrahedron (16, 17). The result of this molecular configuration is the formation of dipoles and the consequent electrostatic attraction through H-bonds. This allows the negatively charged O of one water molecule, to bond with the positively charged H+ of another molecule, establishing interactions between them (16–18) (Figure 1).

### Behavior of water in the human body

The human body is primarily composed of water, accounting for approximately 60% of its total weight. This aqueous percentage is divided into various compartments: around 40% in the intracellular space, 2% in the extracellular space, 15% in the interstitial space, and 5% in the intravascular space (3, 11). Despite having high water requirements, the human body cannot store large quantities, so it needs to acquire water through intake to maintain internal homeostasis. The main source of water comes from fluid intake, estimated at around 1,570 mL per day, and from food, contributing about 675 mL daily. Additionally a limited amount of endogenous water is a result of a reaction product, approximately 300 mL per day, because of the metabolism of macronutrients (3).

### Temperature regulation

Humans can maintain a constant body temperature, regardless of environmental variations, a phenomenon known as homeothermy. The primary source of heat for this regulation comes largely from metabolic processes, although they also receive a minor contribution from external sources of heat, which characterizes endothermy (11, 19). Water plays a crucial role in this process due to its unique molecular properties, such as its high boiling point, melting point, and heat of vaporization. These thermal properties highlight water as a regulator of body temperature by serving as an effective thermal conductor, enabling the absorption and release of heat. The H-bonds present in liquid water can absorb heat, resulting in gradual and gentle changes in body temperature, which act as a protective mechanism for cells against sudden temperature fluctuations (1, 19, 20).

H-bonds confer exceptional properties to water, such as high surface tension, capillarity, heat resistance, high vaporization capacity, and freezing capacity. This translates into a melting point of 0 Celsius (°C), a boiling point of 100°C, and the latent heat of vaporization enthalpy of 2,260 Jouls per gram (J/g), values that exceed those of other solvents. These characteristics result from the strong attractive interactions between water molecules, which provide a notable internal cohesion in its liquid phase. H-bonds are relatively weak, with approximately 10% covalent character and 90% electrostatic character, and they have a bond dissociation energy (the energy required to break the bond) around 23 kilojoules per mole (kJ/mol). This contrasts with the much stronger covalent bonds, such as the O-H bond with a bond dissociation energy of 470 kJ/mol and the carbon (C)-C bond with a bond dissociation energy of 348 kJ/mol (16, 17).

**Figure 2:**
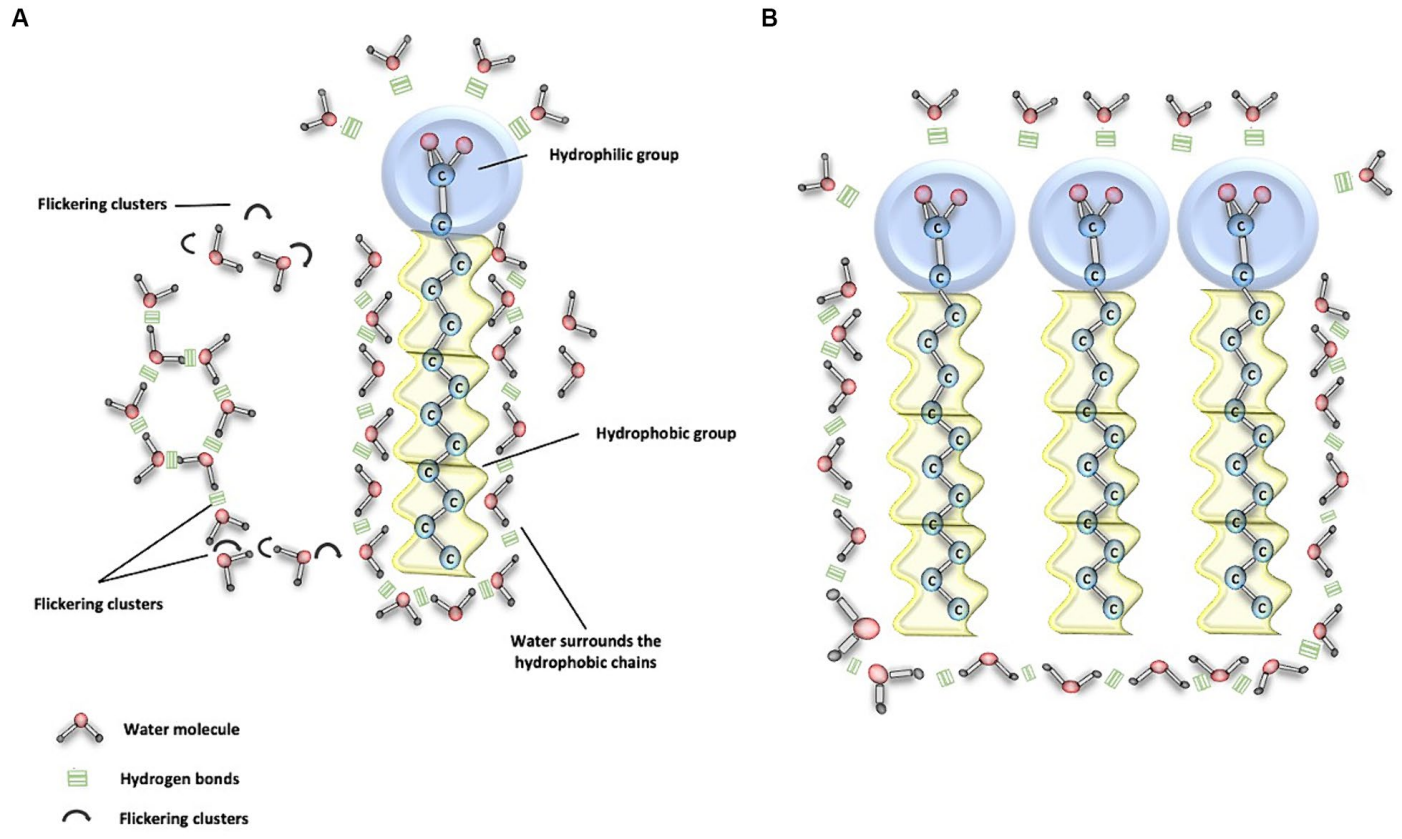
Water and phospholipid interaction. **(A)** This figure demonstrates the interaction between water molecules and the polar heads of phospholipids, depicted as blue circles. Here, water molecules form hydrogen (H) bonds, illustrated by three continuous green lines, facilitating the connection with the polar component. Conversely, the apolar (hydrophobic) tails of phospholipids do not directly engage with water molecules. Nevertheless, the cohesive properties of water enable it to encapsulate the hydrophobic regions, which is essential for the assembly and integrity of phospholipid structures. Moreover, the dynamic nature of water is crucial for transport processes. Transient H-bonded clusters of water molecules, which last for approximately 20 picoseconds, are continually breaking and re-forming, typically in about 1 picosecond. These ephemeral assemblages are known as flickering clusters, and they play a significant role in the fluidity and functionality of cellular membranes. **(B)** This figure illustrates the clustering of various phospholipids as a result of water movement due to its interaction with polar and nonpolar structures in bodily fluids.

In its liquid state at 37°C, water generates thermal energy necessary for the breakage of H-bonds. This process occurs within a time frame of approximately 1 to 20 picoseconds (1 ps = 10^−12^ s). As a result, there is a constant motion of molecules because when one bond breaks, another immediately forms with a different molecule. This phenomenon is known as ‘flickering clusters,’ a term that refers to temporary groupings of hydrogen bonds during the liquid phase of water (16, 17, 21) (Figure 2A). These extensive networks of H-bond connections provide internal cohesion to water, establishing links between solutes that allow interaction and communication over long distances. This confers upon water the ability to absorb heat, which is one of the fundamental mechanisms for body thermoregulation (17, 21).

At higher temperatures, the lifetime of H-bonds is shorter, leading to a continuous motion of water molecules, ultimately resulting in the release of molecules that are loosely bound to each other. This process marks the evaporation phase of water. Conversely, at very low temperatures, molecules remain fixed in space, giving rise to the solid phase known as ice. Before freezing, water expands, resulting in lower density and greater volume compared to water in its liquid state (16, 17).

### The water as a buffer in the human body

Water plays a crucial role as one of the most significant determinants in the acid–base balance of the human body, functioning as a buffering system in various bodily fluids (22). According to the models proposed to understand acid–base disorders, water fulfills a vital function in these processes (23). For instance, in the Henderson-Hasselbalch model, it is established that bicarbonate and carbon dioxide (CO2) are independent variables, and their mutual relationship is the determining factor in pH changes in bodily fluids. Water participates in the formation of carbonic acid and bicarbonate through a reversible reaction with CO2 (23, 24). This reaction (Reaction 1) is facilitated by the enzyme carbonic anhydrase found in various cells and organs, such as erythrocytes, neurons, the intestine, endothelium, and striated muscle, among others (18, 22–24).

$\mathrm{Reaction}\;1:CO2+H2O↔\left[H2CO3^{-}\right]↔\left[{HCO3}^{-}\right]+\left[H^{+}\right]$

where H2O is water, H2CO3- is carbonic acid and HCO3^**−**^ is bicarbonate.

Another model that grants more significance to water is the one proposed by Peter Stewart, which allows for an explanation of acid–base disturbances in critically ill patients that cannot be adequately explained using the Henderson-Hasselbalch model. Stewart posits that the acid–base balance occurs in a solution primarily composed of water, which acts as a solvent and interacts with strong ions, weak acids, and some macromolecules as solutes. This interaction results in the dissociation of water, generating H+ ions or hydroxide ions (OH-), which must adhere to the principle of electroneutrality and maintain mass conservation (22–25).

Water, with a high molar concentration of approximately 55 moles per liter, is 400 times more concentrated than sodium in the body. Possessing this characteristic, it becomes an abundant source of hydrogen ions for biological fluids, enabling it to play a crucial role in the body’s acid–base balance (22, 26). Another important property of water, its electric dipole nature (1, 16, 22). The water molecule carries a negative electric charge on the oxygen atom and two positive charges on the hydrogen atoms. This charge distribution allows water to attract cations (positive ions) and anions (negative ions) from ionic compounds in a process known as ionic solvation. Due to this capacity, water is an excellent solvent for ionic compounds and helps maintain electroneutrality in various bodily fluids. Electroneutrality refers to the balance between positive and negative charges within a system (16, 22, 26).

The water dissociation constant, known as Kw, is very low. Kw has a value of approximately 4.3 × 10–16 millimoles per liter (mmol/L), which means that only about 1 in every 107 water molecules is ionized into H+ and OH- (19). This low dissociation constant accounts for the significant reserve of H+ ions and OH- in water, making it one of the primary buffering systems in the body. Buffering systems are substances capable of responding to abrupt changes in acidity or alkalinity, helping to maintain the body’s acid–base balance (22, 26, 27).

In summary, water’s high molar concentration, electric dipole nature, and low dissociation constant enable it to participate in the body’s acid–base balance as a solvent for ionic compounds and as a buffering system that responds to changes in acidity or alkalinity (22, 26).

### The role of water in physiological solutions

The role of water in numerous physiological processes within the human body is of paramount importance. Its contributions are notable in protein folding and aggregation, interactions among ions, ligands and enzymes, ion transport across membranes, signal transduction, gene expression regulation and the cell cycle (1).

As a solvent, water can establish various covalent interactions, such as electrostatic interactions, van der Waals forces, and solvent-induced interactions (1, 16). In terms of electrostatic interactions, water has the capacity to form bonds with ions through electrostatic forces, allowing it to maintain a balance of major cations and anions in various body compartments. An example of this is water’s ability to dissolve salts like sodium chloride (NaCl), weakening electrostatic interactions through H-bonding and facilitating the dissociation of these molecules (1, 16). This property holds clinical significance as one of the most commonly used intravenous solutions in the management of hospitalized patients is saline solution, primarily composed of Na + and Cl-. When this solution comes into contact with water, these ions dissociate. Na+, being a molecule with high affinity for water (known as kosmotropic), attracts and retains fluid within the intravascular space, resulting in a temporary increase in volume in this space before equilibrating with extracellular fluid. This property underlies the use of intravenous fluids in the processes of hydration and resuscitation for patients in emergency and ICU (4, 28, 29).

On the other hand, there are nonpolar molecules, known as hydrophobic, which exhibit little affinity for water and are unable to establish favorable interactions with water molecules (16). In bodily tissues, this leads water to form organized associations with other molecules through hydrogen bonds, causing hydrophobic components to be surrounded by water molecules (Figure 2B). There are also compounds with both polar and nonpolar regions, known as amphipathic molecules (16, 28). When these compounds interact with water, they facilitate favorable interaction with the polar region, while the nonpolar region tends to avoid contact with water, leading to the organization of these structures into micelles (16). The significance of micelles lies in the absorption and transport of fatty acids and bile, particularly in the digestive tract (16, 28).

**Figure 3:**
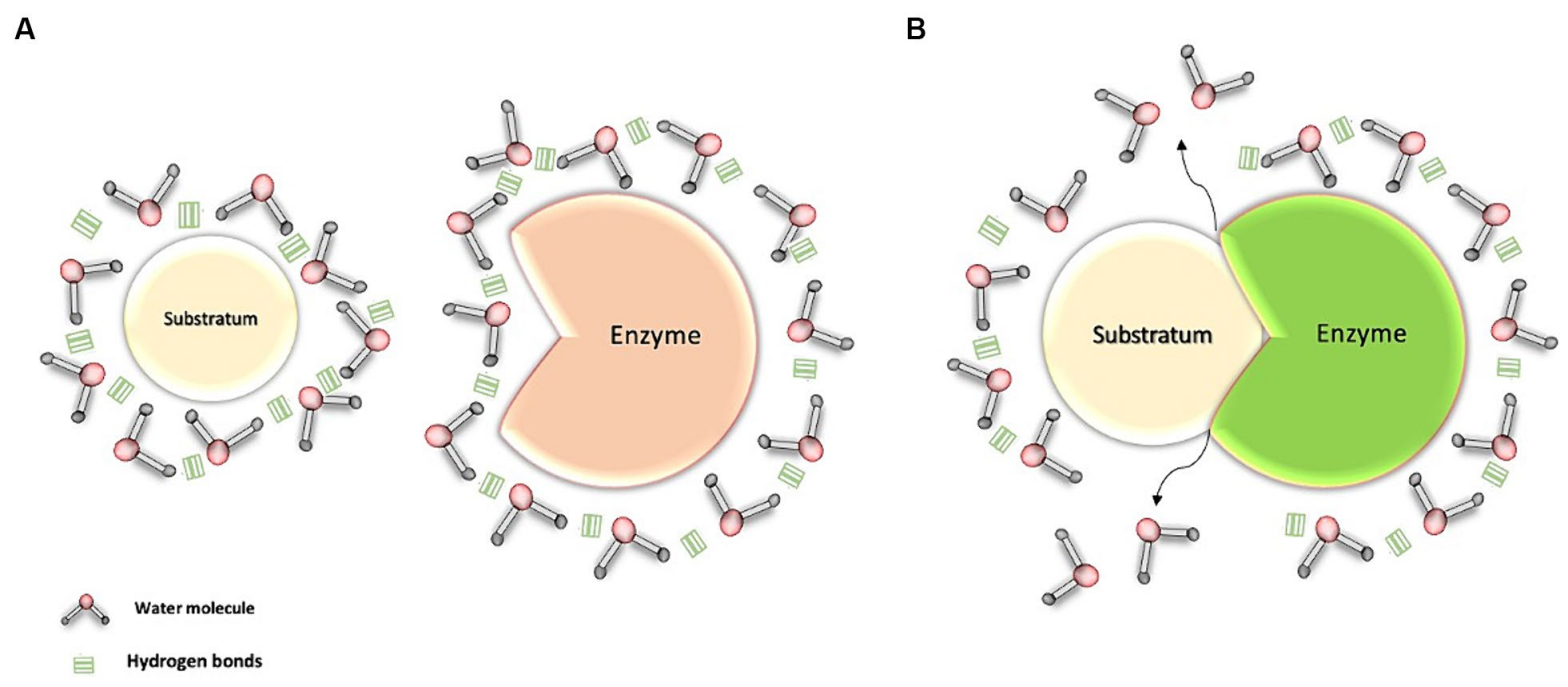
Interaction of water with the enzyme-substrate complex. **(A)** Transport of the enzyme and its substrate through water: Water molecules, through hydrogen bonds, cluster around the substrate and the enzyme, both of which are apolar molecules. **(B)** Interaction between water molecules and the enzyme-substrate complex: This interaction facilitates the movement of these structures within the aqueous environment. As a result, the water molecules promote the formation and stabilization of the enzyme-substrate complex, thereby facilitating its activation.

In our body, various amphipathic biomolecules, such as proteins and phospholipids, play a fundamental role in the structure of biological membranes. These biomolecules have both polar and nonpolar regions, allowing water interaction at both sites (11). In the process of transport and enzyme-substrate interactions, some amphipathic and nonpolar proteins employ transport and clustering mechanisms to reach their respective binding sites in a predominantly aqueous environment (16, 28, 29) (Figure 3).

### Surface tension of water

**Figure 4:**
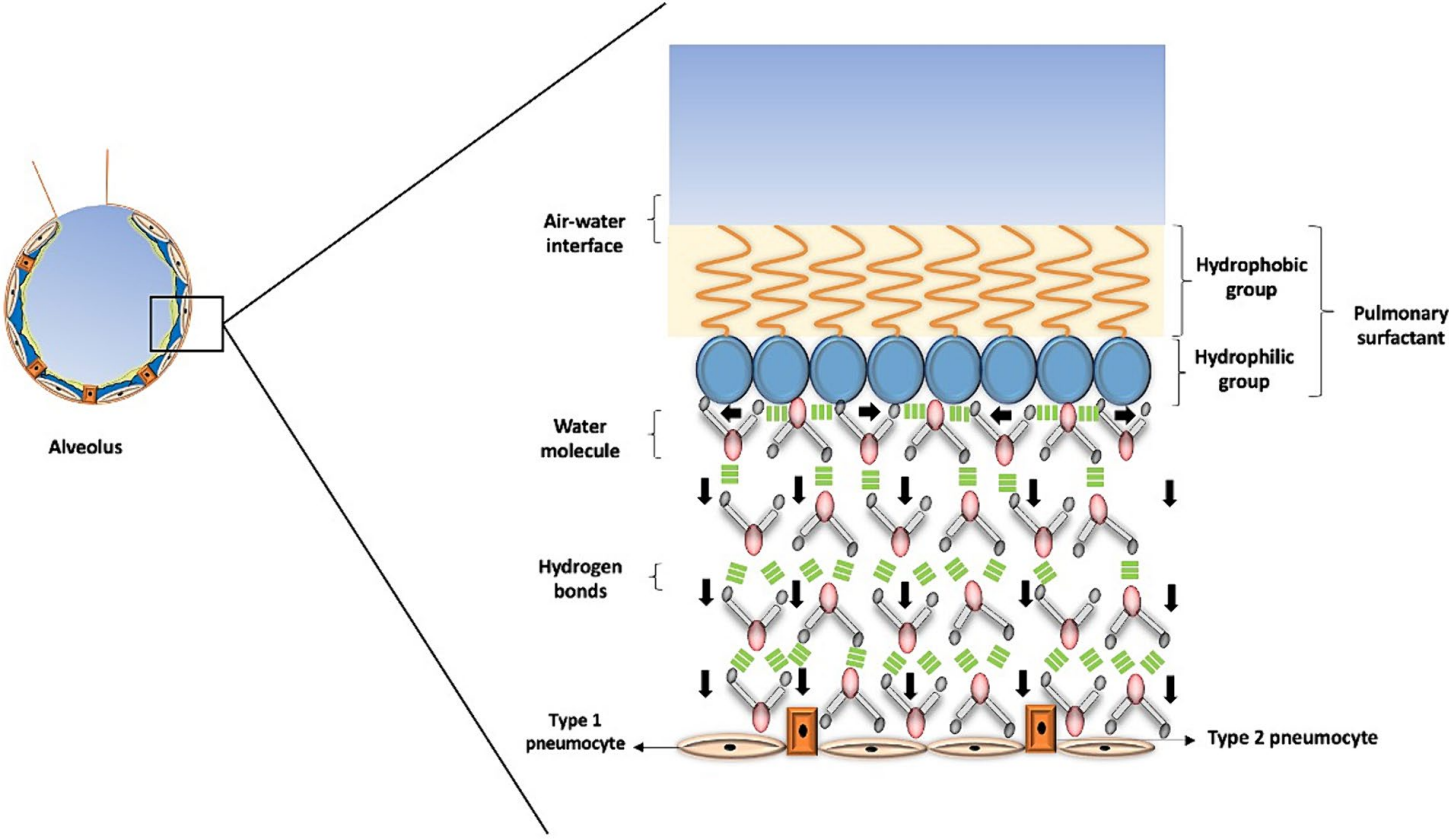
Surface tension in the alveolus. This figure illustrates how surface tension helps prevent alveolar collapse. The pulmonary surfactant has a hydrophobic portion that interacts with air, forming the air-water interface, while its hydrophilic portion contacts water molecules. These molecules form tight bonds that generate horizontal forces, depicted by black arrows pointing horizontally, and a downstream force, represented by black arrows pointing vertically. This interaction produces the necessary surface tension in the alveolus, where the role of water is crucial in preventing alveolar collapse and facilitating gas exchange.

Water assumes a pivotal role in the air-liquid interface within the lungs, thereby influencing the process of lung recoil. Surface tension, a force binding molecules at the air-liquid interface (1, 30), is of paramount importance. The significance of water lies in the fact that the forces among its molecules, while in a liquid state, equilibrate in various directions, ultimately leading to a net force of zero within the liquid. However, at the surface, water molecules are attracted to all other water molecules in the liquid, creating a net force directed inward into the liquid and away from the gaseous phase, leading to the creation of surface tension (31, 32) (Figure 4). In the case of pulmonary alveoli, this results in a surface tension measured at approximately 70 Dyne per centimeter (dynes/cm), which contributes to increased lung recoil and could potentially lead to alveolar collapse (2, 31, 32).

Pulmonary surfactant is a substance that regulates surface tension in the alveoli, reducing it to approximately 25 dynes/cm. This substance consists of a hydrophilic portion that binds to water molecules on the surface and a hydrophobic region that prevents the surfactant from mixing with the liquid phase, keeping it at the air-liquid interface. Several critical conditions are associated with an increase in surface tension, hence an increased risk of alveolar collapse (33). These conditions include acute respiratory distress syndrome (ARDS), pulmonary edema, pneumonia, sleep apnea, pulmonary atelectasis, cystic fibrosis, asthma, among others.

### Water/solute homeostasis in critical care: distribution, sodium balance, and osmosensing

"We come from water, we are largely made of water, water sustains us, and we continually strive to maintain balance with the ocean that surrounds us."

Ricardo Buitrago M.D, Exploratorium Group

Balancing and providing support in the dynamic changes of critically ill patients is a daily challenge for intensivists. In this regard, critically ill patients often face the difficult balance between edema and dehydration, or even both, as pathological states resulting from a loss of internal homeostasis. The question is: how can an appropriate state of hydration be maintained without increasing extracellular volume? This is one of the main challenges in critically ill patients, as it aims to maintain adequate organ perfusion (34, 35).

#### Distribution

Water and its distribution through different compartments determine the volume status (36). The causes of changes in body fluid volumes can be determined by different methods in critical care patients, including anamnesis, physical examination, electrolytes, arterial blood gases and ultrasound (37). There are different compartments of water distribution (intracellular and extracellular spaces) and volume status (euvolemia, hypervolemia and hypovolemia) in therms of physiology and its application in clinical practice, and they assess at the bedside impact on decisions and outcomes (30). Moreover, the distribution of total body water (TBW) through different methods, has been suggested as an indicator of cell membrane function, which can be interpreted as cellular integrity (38).

To carry out adequate management of acute critical illnesses, the evaluation of TBW and its distribution must be taken into account. In terms of TBW, the knowledge of the fluid balance and change in body weight are used and common ways to determine the variation in water balance (39). Two-thirds of TBW (approximately 40% of weight) is within the intracellular space, while the remaining third (20% of weight) corresponds to the extracellular space. Water can be lost in one of these two compartments (11).

Neurological, circulatory symptoms, physical examination, and laboratory tests (serum and urinary sodium, serum creatinine, and plasma urea nitrogen) can help distinguish the affected compartment. A quarter of the extracellular fluids (5% of body weight) are retained in the blood vessels as plasma or effective circulating volume, whose decrease can be assessed through physical examination (blood pressure, pulse rate, jugular venous dilation), if there is invasive monitoring, with filling pressures and cardiac output, or by non-invasive methods such as measuring the diameter of the inferior vena cava by ultrasound or bioelectrical impedance analysis (38).

### Sodium balance

The most prevalent hydroelectrolytic disorder observed in patients ICU is dysnatremia, a condition intricately linked to the likelihood of in-hospital mortality (40–42). Although alterations in Na + levels can be attributed to fluctuations in total-body salt content, the majority of dysnatremic cases stem from an imbalance between water intake and loss. This imbalance profoundly impacts serum Na + levels (42). Taking into account this physiological premise is the reason why the most important aspect when addressing dysnatremia is to accurately determine the volume status of our patient. This entails not only determining the distribution of body water in the various physiological compartments but also evaluating factors such as fluid intake, output, and losses. Additionally, it is crucial to identify any underlying etiologies contributing to the dysnatremia, which may include conditions such as renal dysfunction, endocrine disorders, or medication-related effects (43). Once the volume status and potential underlying causes are elucidated, targeted interventions can be implemented to restore water balance and mitigate the risk of adverse outcomes. These interventions may encompass fluid resuscitation, fluid depletion, electrolyte replacement therapy, to ensure optimal management of dysnatremia and improve patient outcomes (44).

### Osmosensing

Mammals have developed regulatory mechanisms aimed at maintaining plasma osmolality at stable levels (45–47). When abrupt changes in plasma osmolality occur, a series of mechanisms leading to the sensation of thirst and sodium appetite are triggered (48). The organum vasculosum laminae terminalis (OVLT) is considered the primary osmosensory area par excellence in the central nervous system (CNS), housing a group of neurons specialized in osmosensing, a key physiological process for maintaining body water homeostasis (49, 50). Additionally, there are other secondary osmosensory areas in the CNS with similar functions (49, 50). The activity of these osmosensory neurons is mediated by mechanosensitive effects due to changes in tonicity (hypotonic or hypertonic) and compensatory neuronal mechanisms that aim to restore volume by promoting or inhibiting diuresis (51, 52). This is achieved through the transduction of neuronal signals via ion channels on the membrane known as Transient Receptor Potential Vanilloid Type-1 (TRPV1) channels, which may exhibit greater or lesser activity depending on cellular volume changes induced by contraction or depletion in the case of hypertonicity, or inflammation or edema in the case of hypotonicity (53–55). Neuronal response to hypotonicity involves the activation of channels to allow the efflux of Cl-, K+, and water, a process known as regulatory volume decrease; whereas in hypertonicity, Na+, K+, and Cl- absorption is promoted, termed regulatory volume increase (56, 57). The neuronal cytoskeleton plays a significant role in the mechanical activation and deformation of osmosensitive neuron membranes (58–60).

### Thirst in intensive care: a neglected issue

One of the challenges faced by critically ill patients in ICUs is discomfort. According to the Inconforts des Patients de REAnimation (IPREA) study, which assessed discomfort in 34 ICUs in France, thirst was identified as a common issue among these patients. The study’s findings revealed that addressing this problem led to a significant improvement in patient discomfort. Thirst and a lack of privacy were highlighted as the primary causes, with a significant positive impact, showing differences of −1.08 (95% Confidence interval (CI) −1.74 to −0.42, *p* < 0.001) and − 1.08 (95% CI −1.49 to −0.67, *p* < 0.001), respectively (61).

Thirst is one of the most frequently reported symptoms in critically ill patients. The prevalence rates of thirst exceed 70%, with approximately 33 to 52% of patients describing its intensity as moderate to severe, ranging from 18 to 52% (61). For this reason, thirst is a significant issue in the ICU and may even be related to the body’s need to maintain osmolarity balance. However, assessing thirst in the ICU is a challenge for the intensivist due to multiple associated factors, such as delirium, other discomfort-related causes, and patients with invasive mechanical ventilation. In the study conducted by Nascimento et al., a clinical validation of perioperative thirst was performed, and among the 150 postoperative patients evaluated, characteristics related to thirst were identified: dry mouth, dry lips, and desire to drink at 86, 82, and 72%, respectively. Finally, it was identified that the factors most associated with postoperative thirst were dry mouth OR 28.2 (CI 4.35–1196.76) and the use of anticholinergics OR 2.64 (CI 1.01–7.54) (62). Few studies have evaluated the manifestations of thirst in intubated and ICU patients, which is why a validated scale is required to promptly recognize thirst in critical care patients.

The sensation of thirst in critically ill patients results from the interaction of complex mechanisms involving stress factors that produce variations in blood osmolality (normal value: 275–295 mOsm/kg) and inappropriate responses of compensatory mechanisms leading to an increase in osmolarity, intracellular dehydration, and consequently a water deficit (63–66). This results in an alteration in the homeostasis of body water and, consequently, the physiological sensation of thirst. Central osmoreceptors, located in anatomical regions of the central nervous system that lack a blood–brain barrier, are highly vascularized circumventricular organs that normally detect small changes of 1 to 2% in blood osmolality (3 mOsm/kg), promoting the secretion of vasopressin by the neurohypophysis, even before the patient perceives the sensation of thirst (46, 67–70).

Vasopressin acts mainly at the renal level on type 2 vasopressin receptors (V2) in the basolateral membrane of the distal tubules and collecting ducts. V2 receptors, which have 7 transmembrane domains and are coupled to G proteins, activate an intracellular signaling cascade leading to the phosphorylation and activation of type 2 aquaporin channels (AQP-2), increasing water reabsorption and reducing urine volume loss (71). Additionally, imbalances between intracellular and extracellular fluid due to various etiologies such as hemorrhage, vomiting, diarrhea, or sweating, cause hypovolemic thirst (72, 73). Compensatory mechanisms are activated, mainly the Renin-Angiotensin-Aldosterone System (RAAS) and the sympathetic Autonomic Nervous System (ANS), acting at the level of the proximal renal tubules. These mechanisms promote the reabsorption of water and Na + and redistribute them to different compartments, decreasing Na + appetite (74–76).

### Loss of hydrosaline balance

Sodium is the key molecule in regulating the hydrosaline balance in the intravascular space. It can be found in the body in the form of chloride or phosphate. In the management of critically ill patients, various conditions such as the use of loop diuretics, acute kidney injury (AKI), insensible water losses, and diarrhea can lead to states of hypernatremia. These states result from the loss of free water or a decrease in sodium excretion in the urine, leading to a hydromineral imbalance. Additionally, conservative water restriction can also contribute to the development of hypernatremia, which has been associated with increased mortality in ICU (34, 77).

Several studies have shown that the kidney has an inefficient capacity to excrete Na+. In healthy individuals, when Na + intake is increased (approximately 3.2 grams per day), it takes about 5 days for the intake to balance with renal excretion. During this time, Na + exerts an osmotic load, leading to fluid retention in the body. To counteract this osmolal imbalance, the body accumulates free water. This mechanism has an evolutionary justification, as herbivorous animals, omnivores, and humans have limited renal capacity to excrete Na+, promoting water conservation (34, 78).

Certainly, in patients receiving intravenous solutions containing Na^+^ and Cl^−^, a problem can arise due to the kidney’s inability to conserve free water through urine concentration. This process involves high metabolic demand, as it requires the accumulation of urea in the renal medullary interstitium to generate sufficient osmotic force, resulting in increased systemic energy consumption. When the maximum level of Na + in the body is reached (250–300 mmol/L), the kidneys require an intake of free water to increase urine volume and achieve sodium excretion, a phenomenon known as natriuresis (34, 78, 79).

Despite the limited research on hypernatremia in patients treated with free water via enteral tube feeding and its impact on mortality, there are currently no clinical studies comparing the administration of free water via enteral tube feeding as maintenance fluid in critically ill patients without hypernatremia with the use of intravenous isotonic solutions. This emphasizes the need for a better understanding and focus on intravenous fluid management in the ICU (80, 81).

### Hypothermia

In the management of patients in ICUs, the regulation of body temperature has become a crucial aspect. Intravenous administration of physiological solutions can influence the temperature by interacting with the water in the body. When body temperature (T°) is low the water molecule will have less movement due to increased stability and fewer breaks in the hydrogen bonds between molecules, resulting in fewer flickering clusters (16, 17, 21). The administration of warm intravenous fluids contributes to heat absorption by the water in the internal environment, breaking hydrogen bonds and raising the temperature (1, 11, 19). In the case of severe hypothermia, defined as a core body temperature below 28°C, clinical manifestations can include delirium, hallucinations, coma, bradycardia, or arrhythmias (atrial fibrillation, ventricular fibrillation, asystole) at 20°C. In moderate hypothermia, typical symptoms include confusion, lethargy, arrhythmias, tachypnea, bradypnea, reduced antidiuretic hormone (ADH) secretion, renal tubular acidosis, hemoconcentration, and impaired platelet function (20).

### Hyperthermia

In situations where body temperature increases, such as in the case of fever, a decrease in T° can be achieved through heat absorption via hydrogen bonds, evaporation in the respiratory passages, and the elimination of fluids through diuresis and sweating (1, 11, 19). As body temperature rises, water molecules will exhibit increased movement due to a greater disruption of hydrogen bonds and a higher number of flickering clusters (16, 17). In cases of severe temperature dysregulation deleterious cellular events can transpire, encompassing damage to the cell membrane, mitochondria, and protein denaturation, potentially culminating in cellular demise. Furthermore, local effects may ensue, such as the release of cytokines and inflammatory mediators, vascular stasis, extravasation, and the development of edema. These events can exert systemic repercussions, including endotoxemia and bacterial translocation from the intestinal microenvironment (19, 27).

In case of hyperthermia may present with delirium, hallucinations, seizures, coma, tachycardia, heart failure, myocardial, hepatic, and renal injury, myoglobinuria, and disseminated intravascular coagulation (19, 82). Therefore, one of the primary goals in the ICU is to maintain appropriate thermoregulation in the patient, tailored to their specific pathology.

**Figure 5:**
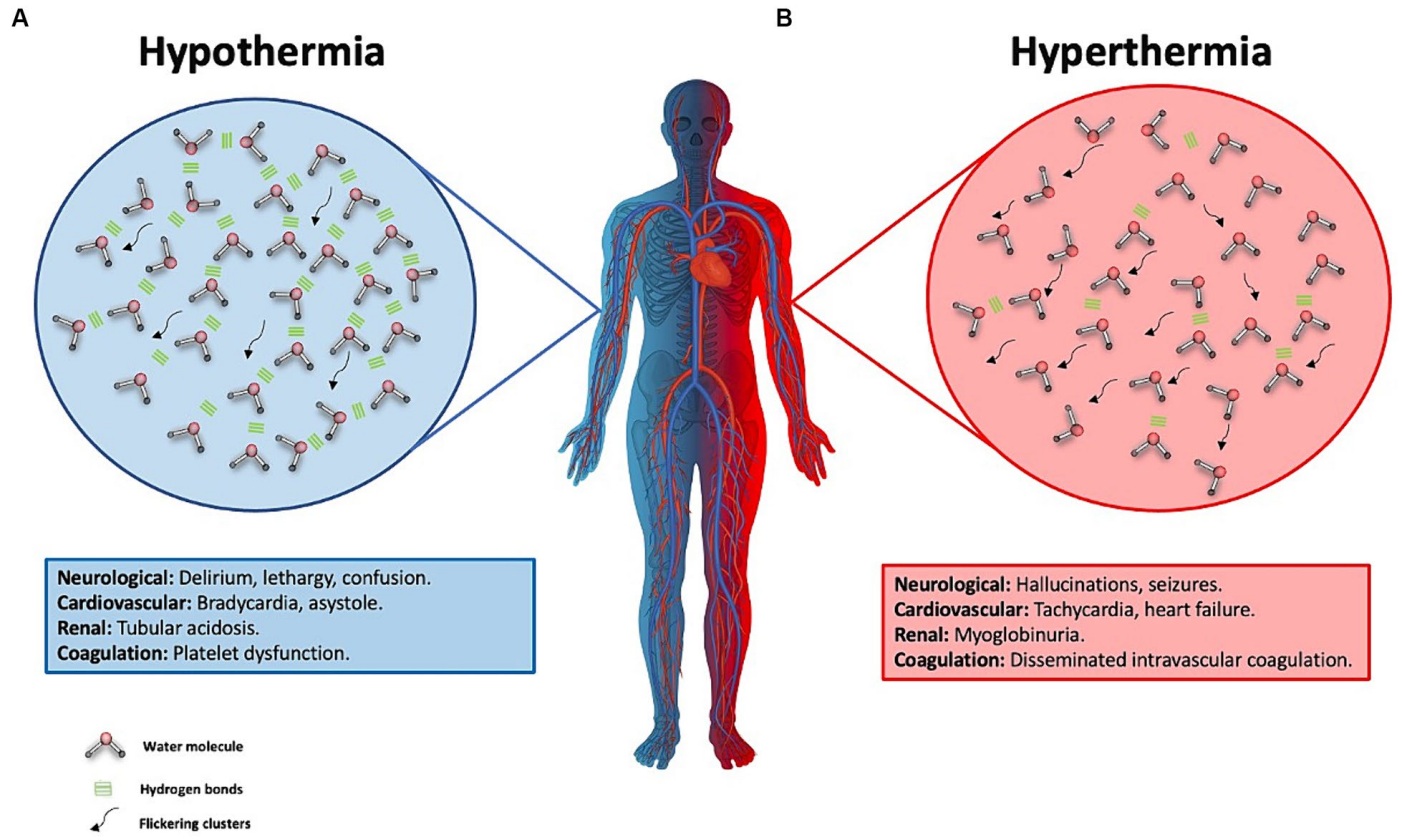
Water behavior in hypothermia and hyperthermia and its effect on the human body. **(A)** This figure illustrates hypothermia, represented by one half of the body in blue. The background blue circle depicts water molecules with more stable hydrogen bonds, indicating reduced water molecule movement due to a longer half-life of these hydrogen bonds. These are known as flickering clusters, which are represented by the black arrows. Clinical manifestations of severe hypothermia, defined as a body temperature below 28°C, are described in the blue box. **(B)** Hyperthermia is represented by half of the body colored red and a circle of the same color, showing water molecules in constant motion. This is due to a decrease in the number of hydrogen bonds, resulting from their greater disruption, which facilitates increased molecular movement and a higher count of flickering clusters. These clusters are illustrated by black arrows, symbolizing intensified molecular dynamics under conditions of elevated body temperature. The clinical manifestations of hyperthermia are detailed in the red-background box.

The T° changes associated with the administration of intravenous fluids are intricately linked to the cardiac output of the patient. For every 4 liters of saline solution administered, there may be a corresponding increase of 1°C in the patient’s core body temperature.This, in turn, leads to an elevation in the heart rate by approximately 4.4 beats per minute (bpm) (19, 82). It has been demonstrated that maintaining a core body temperature above 39.5°C is associated with increased mortality (82). In patients who have suffered a cardiac arrest, every 1°C increase above 37.7°C is linked to higher mortality within the first 48 h (83). In the case of patients with traumatic brain injury, reducing core body temperature can decrease cerebral metabolism by up to 6% for every 1°C decrease, resulting in reduced cellular damage (83–88) (Figure 5).

### Water-related illness in the ICU: an underestimated problem in the ICU

It is a widely recognized fact within the medical community that fluid overload in critically ill patients correlates directly with a higher mortality rate (89). Medical professionals in critical care often encounter extraordinarily complex situations regarding the fluid balance of their patients. This challenge is exacerbated by the variability of pathologies that can coexist in the same individual, such as pulmonary edema, renal hypoperfusion, capillary leak, and anasarca, among others. It is essential to understand that addressing these conditions in isolation would be a limited approach, as the simultaneous presence of multiple signs suggests a more complex underlying alteration in the body’s fluid balance, which can be considered as “Water Disease.” This water disease arises because of the loss of previously described fluid regulatory mechanisms, underscoring the need for a comprehensive and multifaceted approach in its clinical management.

During the initial phases of resuscitation, large volumes of isotonic fluids are administered intravenously. However, studies have shown that following a liberal fluid administration strategy can increase the mortality rate in these patients. Consequently, the current trend is to adopt a fluid restriction strategy (5, 34, 90). However, it is important to note that this strategy can lead to a loss of free water in the body, resulting in higher concentrations of blood components and cellular dehydration (4, 35, 90).

**Figure 6:**
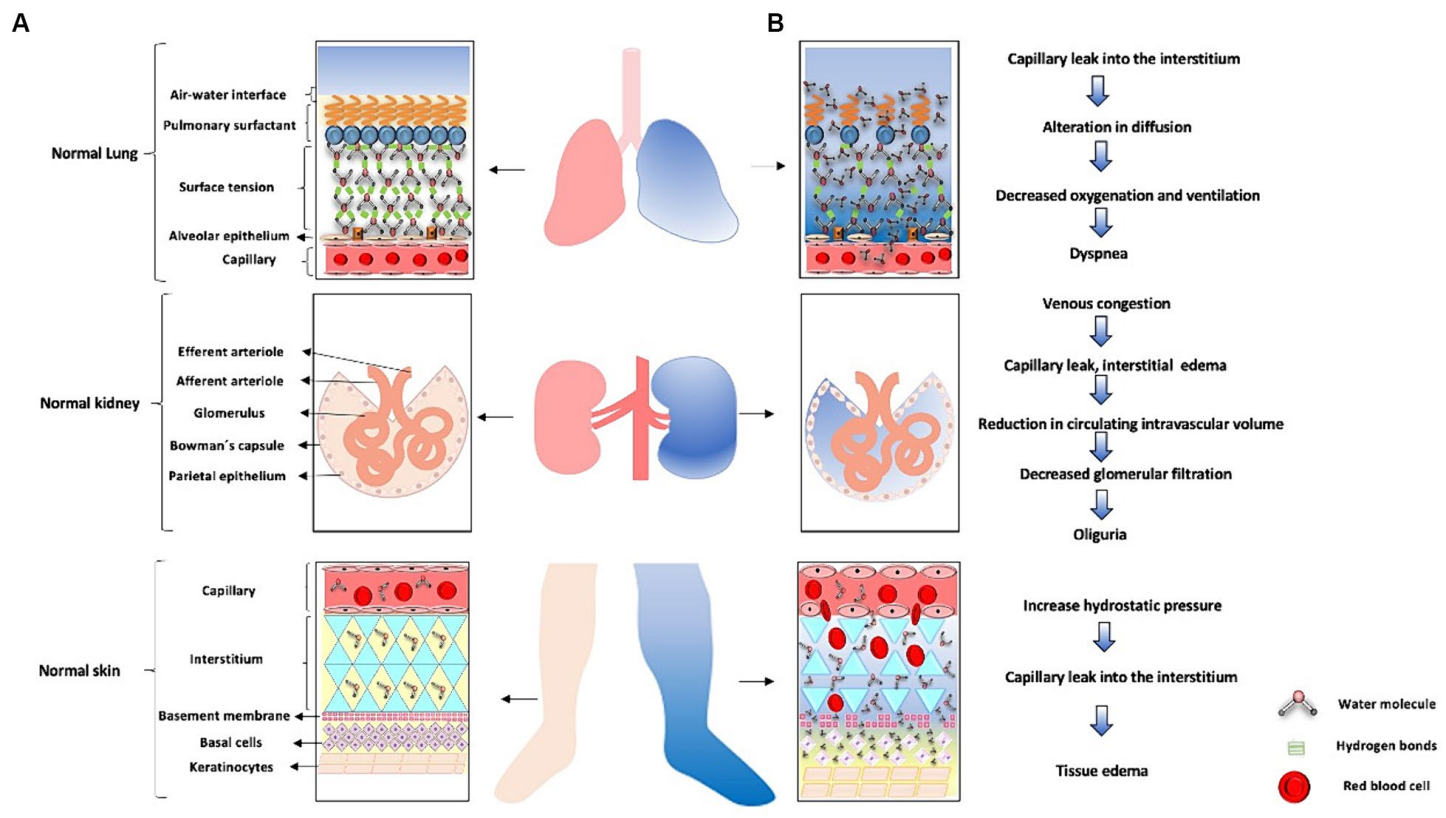
Water-related disease in the ICU. **(A)** The lung is shown in pink with a normal alveolar-capillary membrane. Next, a healthy kidney with intact nephrons is depicted. Finally the skin, represented as the light pink lower limb and its respective normal structures on the left side of the figure. **(B)** Lung whith blue-colored represents excess water manifested as pulmonary edema secondary to loss of alveolocapillary membrane function. When intercellular junctions of the endothelium are disrupted, water molecules shift, leading to loss of surface tension and ultimately pulmonary edema. On the other hand, when there is an excess of fluids due to regulatory loss, venous congestion occurs, as represented by the blue-colored kidney and nephron on the right side. The excess water leads to venous congestion, leading to capillary leakage with subsequent interstitial edema, loss of intravascular volume, causing a decrease in renal perfusion pressure and subsequently acute kidney injury. The right lower limb in blue represents excess water leading to tissue edema. When there is loss of intercellular junctions of the endothelium due to increased hydrostatic pressure, there will be capillary leakage with extravasation of water molecules into the interstitial space, resulting in tissue edema.

**Figure 7:**
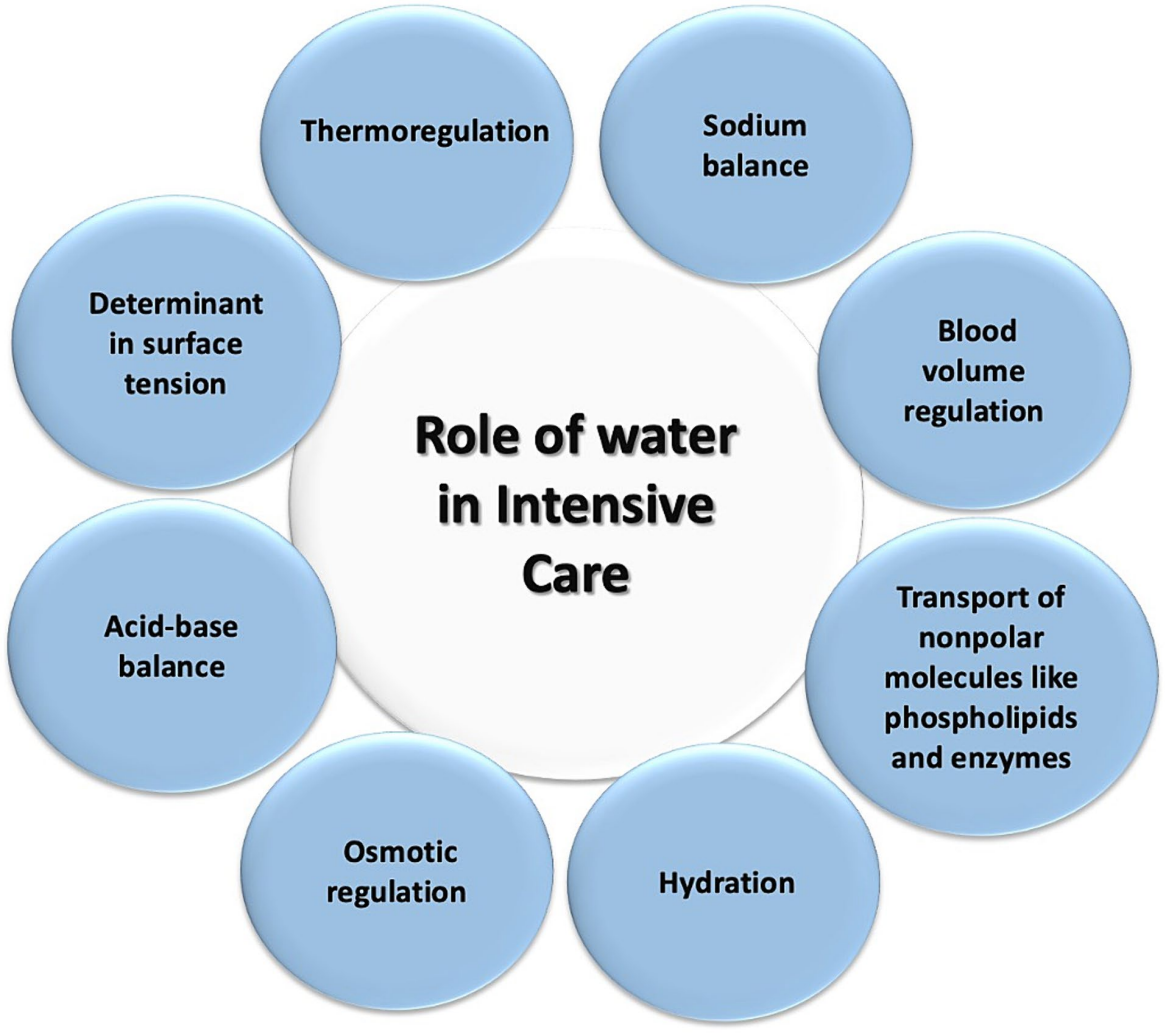
Role of water in intensive care. This diagram illustrates the various processes in which water plays a role for patients in the intensive care unit.

In patients experiencing a state of shock, there is a redistribution of TBW and intravascular blood volume (91). Furthermore, the use of vasoactive agents like norepinephrine contributes to reduced blood flow in the superior mesenteric artery and the gastrointestinal tract microcirculation, such as in the jejunal mucosa, where a decrease of approximately 70% is observed (91, 92). In shock patients requiring vasopressor agents, this can lead to enterocyte impairment, the cells lining the gastrointestinal tract, in approximately 50% of cases, resulting in gastrointestinal injury (93). Water disease can manifest in various ways in the ICU, affecting multiple systems. An example of this is the respiratory system, specifically in the lungs, where an excess of fluid or loss of integrity in the alveolocapillary barrier can lead to pulmonary edema, resulting in gas diffusion impairment, decreased oxygenation, and ventilation. Another system that can be affected is the renal system, where an acute excess of water can lead to venous congestion, causing increased pressures and damage to the glycocalyx, leading to endothelial damage and subsequent capillary leakage, interstitial edema, decreased intravascular volume, decreased renal perfusion pressure, decreased glomerular filtration rate, and ultimately AKI (94). Lastly, the integumentary system is affected by water disease through the same mechanism of venous congestion, capillary leakage, and extravasation of water into the interstitial space (95). Figure 6 illustrates these systems affected by water disease. Figure 7 summarizes the role of water in the ICU.

## Conclusion

Water, as a universal solvent and a primary component of the human body, stands out for its ability to exist in three states of matter. Its significance is reflected in essential biomolecular processes, such as thermoregulation, alveolar surface tension function, acid–base balance, and osmolarity, among others. In the clinical setting of critical care, supplying water according to the clinical condition is crucial, regardless of the patient’s manifestation of thirst. The complexity of these interactions, linked to various stressors and the inadequacy of compensatory mechanisms, underscores the need for a rational approach to fluid therapy by intensivists. This approach aims to prevent the perpetuation of pathological conditions such as edema and dehydration in situations of homeostasis loss and endothelial dysfunction. Knowledge of the physiology of water leads to the proper management of critical care patients.

## Author contributions

HR-A: Conceptualization, Formal analysis, Funding acquisition, Investigation, Methodology, Project administration, Resources, Supervision, Validation, Visualization, Writing – original draft, Writing – review & editing. AQ-A: Formal analysis, Funding acquisition, Investigation, Resources, Supervision, Validation, Visualization, Writing – review & editing. CF-N: Formal analysis, Investigation, Resources, Supervision, Validation, Visualization, Writing – review & editing. IS-P: Formal Analysis, Investigation, Resources, Validation, Visualization, Writing – review & editing. AS-S: Conceptualization, Funding acquisition, Methodology, Resources, Validation, Visualization, Writing – original draft, Writing – review & editing. RB-B: Conceptualization, Data curation, Resources, Supervision, Validation, Visualization, Writing – original draft, Writing – review & editing. JC-B: Resources, Supervision, Validation, Visualization, Writing – review & editing.

## Glossary

**Table 1:** 

ADH	Antidiuretic hormone
AKI	Acute Kidney Injury
ANS	Autonomic System
AQP-2	Aquaporin-2 channels
ARDS	Acute Respiratory Distress Syndrome
C	Carbon
Ca	Calcium
CI	Confidence interval
Cl-	Chlorine
CNS	Central nervous system
CO2	Carbon dioxide
Dyne/cm	Dyne per centimeter
H	Hydrogen
H +	Positive electrical charge on the hydrogen atoms.
H2CO3-	Carbonic acid
H2O	Water
HCO3^**−**^	Bicarbonate
ICU	Intensive Care Unit
ICUs	Intensive Care Units
IPREA study	Inconforts des Patients de REAnimation study
J/g	Jouls per gram
kJ/mol	Kilojoules per mole
K +	Potassium
mmol/L	Millimoles per liter
Mg +	Magnesium
mOsm/kg	MilliOsmoles per kilogram
Na +	Sodium
NaCl	Sodium chloride
OH-	Hydroxide ions
O2-	Negative electrical charge on the oxygen
OVLT	organum vasculosum laminae terminalis
PS	Picoseconds
RAAS	Renin-Angiotensin-Aldosterone System
SO	Sulfur oxide
TBW	Total Body Water
T	Temperature
TRPV1	Transient Receptor Potential Vanilloid Type-1 channels
UV	Intense Ultraviolet
V2 receptors	Type 2 receptors for vasopressin
